# Epidermal barrier disorders and corneodesmosome defects

**DOI:** 10.1007/s00441-014-2019-1

**Published:** 2014-11-07

**Authors:** Marek Haftek

**Affiliations:** EA4169 “Fundamental, Clinical and Therapeutic Aspects of the Skin Barrier Function”, Université Lyon 1, 8 Avenue Rockefeller, 69373 Lyon, France

**Keywords:** Corneodesmosome, Stratum corneum, Desquamation, Epidermal barrier function, Skin disease

## Abstract

Corneodesmosomes are modified desmosomes present in the stratum corneum (SC). They are crucial for SC cohesion and, thus, constitute one of the pivotal elements of the functional protective barrier of human skin. Expression of corneodesmosomes and, notably, the process of their degradation are probably altered during several dermatoses leading to the disruption of the permeability barrier or to abnormal, often compensative, SC accumulation. These different situations are reviewed in the present paper.

## Introduction

The final product of epidermal differentiation, the stratum corneum (SC), constitutes a barrier that efficiently separates the body’s internal milieu from the terrestrial environment. It is composed of 10–20 layers of dead cornified cells embedded in a highly hydrophobic extracellular matrix. Quasi equimolar proportions of three lipid families, i.e., ceramides, cholesterol and free fatty acids, are necessary for the adequate molecular organization of extracellular spaces and the resulting relative impermeability to water and other substances (Bouwstra et al. 2003; Feingold and Elias 2014). The physical barrier of SC is highly interactive in terms of its constant response to changing environmental conditions and insults. Such a rapid adaptation is possible because of perpetual epidermal renewal accompanied by relatively rapid SC recycling, with a turnover time of approximately two weeks (Hoath and Leahy 2003; Elias and Choi 2005; Haftek 2014). A secondary barrier composed of epidermal tight junctions is located in the stratum granulosum and appears to play an important role in SC formation, notably in the case of the acute abrogation of the principal SC fence (Abdayem et al. 2014; for a review, see the following paper by J.M. Brandner [2015]).

Although SC barrier function depends greatly upon its biochemical composition, no effective barrier would exist without the appropriate tissue structure. The flattened cornified keratinocytes, namely the corneocytes, are delineated by highly insoluble cornified envelopes together with equally cross-linked lipid envelopes. The latter are constituted by a monolayer of ceramides that replace plasma membranes of the living cells. Lipid envelopes constitute the scaffold for the molecular arrangement of extracellular lipids to form stacked bilayer sheets in inter-corneocyte spaces. This layered lipid structure is essential for providing an adequate degree of waterproofing and the SC permeability barrier (van Smeden et al. 2014). Corneocytes remain connected via cell-cell junctions persisting in the SC and their desquamation at the top of the skin depends on the gradual degradation of these cell attachments (Haftek et al. 2011; Haftek 2014; Ishida-Yamamoto and Igawa 2014; for a review, see the previous paper by A. Ishida-Yamamoto [2015]).

**Fig. 1:**
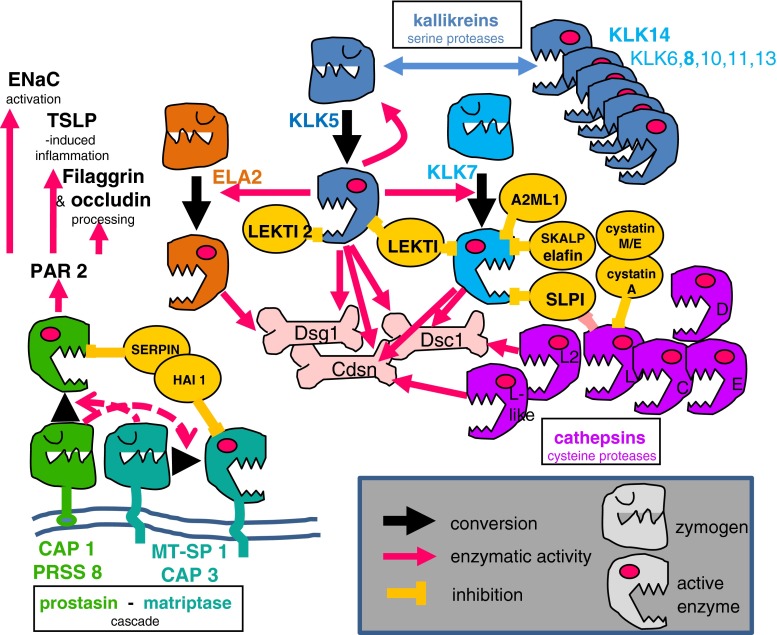
Interplay of proteolytic enzymes with their inhibitors taking place in human epidermis. Intercellular proteins of corneodesmosome are substrates for serine proteases kallikreins (*KLK*), elastase 2 (*ELA2*) and cysteine proteases cathepsins. Plasma-membrane-attached serine proteases of matriptase/MT-SP1/CAP3—prostasin/CAP1/PRSS8 cascade can cross-activate and act through the protease activated receptor 2 on filaggrin and occludin processing, the thymic stromal lymphopoietin (*TSLP*)–mediated adaptive inflammatory response and epithelial sodium channel (*ENaC*) activation. Enzyme activities are tempered by several specific inhibitors. All these interactions are crucial for the maintenance of epidermal homeostasis and stratum corneum barrier function

The principal “mechanical” junctions of the SC, namely the corneodesmosomes, are modified desmosomes from the uppermost nucleated epidermal layers. They retain the molecular composition of the stratum granulosum junctions, notably desmosomal cadherins characteristic of differentiated keratinocytes, i.e., desmoglein 1 and desmocollin 1 but are immobilized at the cell periphery through an extensive enzymatic cross-linking mediated by transglutaminases 1, 3 and 5 (Haftek et al. 1991; Hitomi 2005). Shortly before cornification, the keratinocytes of the granular layer synthesize and excrete into the extracellular spaces a new glycoprotein, namely corneodesmosin, which spontaneously embeds within the intercellular portions of the stratum granulosum desmosomes occupied by cadherins (Serre et al. 1991; Haftek et al. 1997). Corneodesmosin reinforces the junctions and must be degraded by proteases, together with the desmosomal cadherins, to permit desquamation (Simon et al. 2001a; Jonca et al. 2002). A complex interplay of serine proteases (kallikreins) and cysteine proteases (cathepsins) with their respective inhibitors (all excreted through the same vesiculo-tubular system of lamellar granules as the intercellular lipids) is orchestrated by the modifications of SC pH and hydration to result in the progressive digestion of the corneodesmosomes (Haftek et al. 1998; Denda et al. 1998; Hachem et al. 2003; Caubet et al. 2004; Rawlings and Voegeli 2013; Fig. 1). During this degradation process, first the junctions from between the consecutive layers of corneocytes disappear, leaving intact the lateral cell-cell attachments. This results in the subdivision of the SC into a highly cohesive part, the SC compactum, with corneodesmosomes all around the cells and the SC disjunctum, with side-to-side cell connexions only. Once again, the peculiar spatial regulation of this desquamation process might be dependent on structural features: in this case, the persistence of strategically located fusions between the adjacent cornified cell envelopes, i.e., cross-linked remnants of tight junctions (Haftek et al. 2011; Igawa et al. 2011). Functional consequences of this situation can be measured based on the energy necessary for intercellular SC delamination. Indeed, such energy values diminish together with the lowering of corneodesmosome density from the deeper parts of the SC towards the surface (Wu et al. 2006). Kallikrein-7-induced corneodesmosome degradation largely contributes to this process (Levi et al. 2008). The balance between the SC formation and desquamation impacts in an evident way on SC thickness and its barrier function. In many cases, reactive hyperkeratosis reflects a “routine” compensatory response of the epidermis to functional or physical barrier disruption.

## Inherited forms of corneodesmosome dysfunction and their impact on the SC barrier

### Primary defects of corneodesmosomes

Homozygous nonsense mutations in the corneodesmosin (*CDSN*) gene leading to the complete absence of the encoded protein or to residual expression of small non-functional fragments result in peeling skin disease (PSD), a generalized form of peeling skin syndrome, classified as inflammatory form B (MIM270300; Oji et al. 2010; Israeli et al. 2011; Mazereeuw-Hautier et al. 2011; Mallet et al. 2013). In this pathology, the extracellular portions of the corneodesmosomes prove less resistant to mechanical stress and are easily cleaved, especially at the bottom of the SC, at the interface with the granular layer. This mechanical separation of the entire sheet of full thickness SC leaves largely denuded areas with practically no barrier at all. Rupture of the permeability barrier results, in turn, in cytokine production by keratinocytes (Wood et al. 1992) and in an inflammatory reaction typical of this clinical form. A rescue response of the uppermost viable epidermal layers aimed at the re-establishment of the SC barrier is also induced. Most probably, it comprises lamellar granule/lipid release, as suggested by the mouse experiments of the Elias group (Menon et al. 1992) and the up-regulation of the tight junction structures, as documented by a significant increase in the tight junction remnants persisting in the SC of PSD (Haftek et al. 2012). In contrast, specific dominant *CDSN* mutations are associated with autosomal dominant hypotrichosis simplex (MIM146520; Levy-Nissenbaum et al. 2003). In this disease, the truncated mutant corneodesmosin has been found to exert a toxic effect on hair follicles through the formation of amyloid deposits (Caubet et al. 2010).

PSD must be differentiated from another generalized but non-inflammatory form A, the etiology of which has been recently linked to a mutation in *CHST8* gene encoding a Golgi sulfotransferase (Cabral et al. 2012) and from the acral form of peeling skin syndrome. Indeed, the latter constitutes another heterogeneous group of dermatoses (Krunic et al. 2013) with its major variant being caused by mutations in *TGM5*, encoding transglutaminase 5 (MIM609796; Cassidy et al. 2005; Szczecinska et al. 2014).

Homozygous bi-allelic mutations of the desmoglein 1 gene (*DSG1*) observed in rare consanguineous families also impact corneodesmosome function. They result in severe dermatitis, multiple allergies and metabolic wasting syndrome (MIM 615508; Samuelov et al. 2013), although cases without metabolic wasting have also been observed (Has et al. 2014). Although the main structural changes, such as irregular desmosome distribution, hypergranulosis with focal absence of the granular layer and widespread acantholysis within the stratum spinosum and granulosum, result in subcorneal and intragranular separation, a modified SC is also observed showing mixed ortho- and parakeratosis. Corneodesmosome distribution also remains uneven and the disorder is characterized by compromised barrier function, which may expose the immune system to abnormal stimulation and lead to multiple allergies.

As can be logically predicted, analogical SC defects involving the other desmosomal cadherin engaged in the process of SC cohesion, desmocollin 1, will also be discovered with time.

### Protease and protease inhibitor dysfunctions

The protease-antiprotease system efficiently regulates the normal process of SC formation and desquamation (Egelrud 2000; Rawlings and Voegeli 2013). Observation of several pathological states in man and in rodents has helped partially to unravel these complex interactions. However, whether a common pathway and a coordinated regulation of their activity are involved in the terminal differentiation of epidermal keratinocytes remains unclear.

Netherton syndrome is an autosomal recessive genodermatosis (MIM 256500) characterized by congenital ichthyosiform erythroderma, invaginated distal hair shafts and atopic disease. It is caused by mutations in the *SPINK5* gene encoding LEKTI 1, a serine protease inhibitor (Hovnanian 2013). Specific and measured neutralization of kallikreins 5, 7 and 14 by LEKTI is necessary for the limitation of corneodesmosome degradation and hence, in Netherton syndrome, premature desquamation occurs associated with inflammatory reaction and severe barrier impairment leading to multiple allergies (Deraison et al. 2007).

An inverse pathomechanism takes place in recessive X-linked ichthyosis (XLI, MIM 308100) in which deletions in the steroid sulfatase (*STS*) gene result in insufficiency of the enzyme and SC retention (Elias et al. 2004). Steroid sulfatase is necessary for the conversion of cholesterol sulfate to cholesterol, a fundamental building brick of the intercellular lipid lamellae of SC. In XLI, reduction in the cholesterol molecules within the horny layer and the accumulation of cholesterol sulfate provoke disequilibrium in the extracellular lipid species leading to phase separation and suboptimal barrier function. Moreover, the accumulation of cholesterol sulfate substrate (up to 20-fold that of normal values) has been revealed to be a potent inhibitor of SC kallikreins in vitro (Sato et al. 1998) and is thus able significantly to slow down corneodesmosome degradation. Other mechanisms leading to corneodesmosome retention in XLI include (1) low SC pH, out of the neutral to basic operating optima of kallikreins (Ohman and Vahlquist 1998) and (2) the increased presence of Ca^2+^ in the intercellular domains of the lower part of ichthyotic SC possibly contributing to the stabilization of corneodesmosome attachments (Elias et al. 2004).

Autosomal recessive ichthyosis with hypotrichosis (ARIH, OMIM 610765), an inherited disorder linked to mutations in the *ST14* gene coding for a serine protease matriptase, is characterized by the absence of the proteolytic activity of this type II transmembrane enzyme (Chen et al. 2014). Histologically, impaired corneodesmosome degradation, acanthosis and SC accumulation can be observed (Basel-Vanagaite et al. 2007). Another inherited disease linked to various kinds of mutation of the matriptase gene and resulting in the total loss of the expression of the protein is IFAH (ichthyosis, follicular atrophoderma, hypotrichosis and hypohidrosis; OMIM 602400; Chen et al. 2014). The SC barrier defect observed in this latter affliction has been associated with the impaired processing of profilaggrin (Alef et al. 2009). Clinical differences in phenotype between ARIH and IFAH can possibly be explained by the presence of modified matriptase fragments in the former, in the light of evidence that reciprocal cross-activation of zymogen forms of matriptase and its downstream partner prostasin/PRSS8/CAP-1 occurs and is independent of the activation state of the enzymes (Friis et al. 2013). Although the epidermal distribution of human and rodent matriptase diverges significantly (Chen et al. 2014), we should note that reduced filaggrin formation from its profilaggrin precursor has also been reported in matriptase knockout (KO) mice, additionally impacting SC lipid matrix formation and cornified envelope morphogenesis (List et al. 2003). Together, these observations point to a role of the matriptase-activated cascade spanning from keratinocyte proliferation, through their terminal differentiation, to desquamation.

Interestingly, impaired filaggrin processing has been reported in mice models mimicking the human autosomal recessive congenital ichthyosis group of diseases (ARCI; Jobard et al. 2002) through an altered function of arachidonic acid converting enzymes such as 12R-lipoxygenase (Epp et al. 2007). Because such enzymes have no obvious roles in the processing of profilaggrin, defects in the epidermal differentiation processes, even at early stages of differentiation, could affect downstream filaggrin processing.

### Impact of filaggrin mutations and changes attributable to abnormal epidermal differentiation

**Fig. 2:**
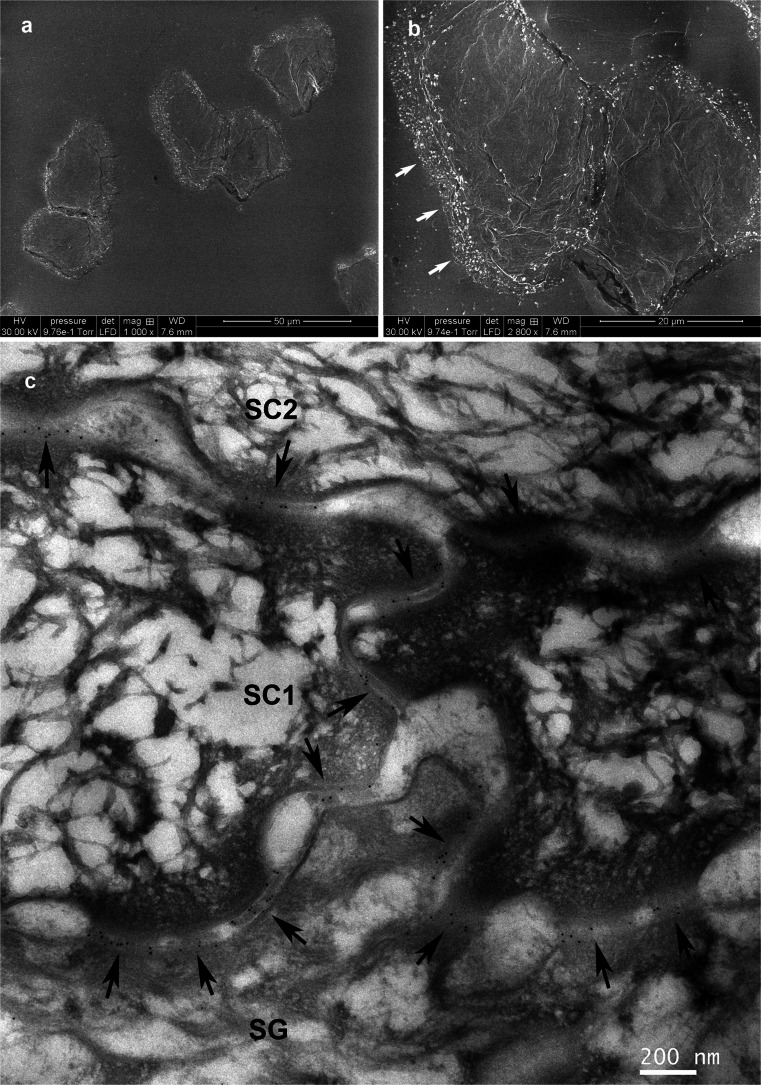
Corneodesmosome distribution as highlighted by immunogold labeling with an anti-corneodesmosin monoclonal antibody. **a**, **b** Labeling of native superficial tape-stripped corneocytes revealed with 1-nm immunogold enhanced with silver coating and observed by scanning electron microscopy. Only lateral rims of the desquamating cells are labeled (*arrows*). **c** Post-embedding labeling with 10-nm gold granules on a vertical section of normal stratum corneum compactum as visualized by transmission electron microscopy (*arrows* corneodesmosomes present both at the lateral and at the ventral/dorsal faces of the cells, *SG* stratum granulosum, *SC1*, *SC2* successive horny layers). *Bars* 50 μm (**a**), 20 μm (**b**), 200 nm (**c**)

Ichthyosis vulgaris (MIM 146700) and atopic dermatitis (ATOD2; MIM 605803) can be associated with loss-of-function mutations in the filaggrin gene (*FLG*; Smith et al. 2006; Palmer et al. 2006; Weidinger et al. 2006). As discussed previously, no straightforward explanation has been proposed for the pathological mechanism involved in which low filaggrin results in impaired barrier function. Nevertheless, recent experimental data suggest the existence of a feedback mechanism involving the N-terminal fragment of filaggrin, which would thus be responsible for controlling epidermal homeostasis (Aho et al. 2012). Free amino acids originating from filaggrin degradation are the main contributors to the NMF (natural moisturizing factor) of the SC. Therefore, the absence or largely reduced presence of filaggrin should impact NMF quantities and, consequently, SC hydration (Dapic et al. 2013). Because filaggrin is also incorporated into cornified envelopes (Simon et al. 1996), one can hypothesize that low filaggrin levels influence the quality of these structures and, in this way, modify the fate of the intercellular lipid lamellae and corneodesmosomes. Indeed, an abnormal distribution of corneodesmosome proteins persisting on the flat lower/ventral sides of superficial corneocytes has been reported in atopic dermatitis, mostly in skin lesions and to a lower extent in non-involved skin (Igawa et al. 2013). This is in stark contrast to the strips from normal subjects in which staining is present only at the lateral rims of corneocytes (Oyama et al. 2010; Igawa et al. 2013; Singh et al. 2014; Fig. 2) and with PSD in which corneodesmosin is not detected. Patterns of corneodesmosome distribution assessed with immunofluorescence by using antibodies to corneodesmosin, desmoglein 1 and desmocollin 1 are similar in atopic dermatitis and in ichthyosis vulgaris, although no information has been provided concerning the eventual existence of *FLG* mutations in the former. This has confirmed the presence of wide-ranging defects in the cornification occurring in atopic dermatitis (Guttman-Yassky et al. 2009) but, unfortunately, does not permit any connection to be made between them and the eventual occurrence of a filaggrin defect. By the way, in Netherton syndrome, which also presents a defective SC barrier function and atopy, the staining pattern is similar, although the corneocytes were stripped in an irregular way. The method of immunofluorescent labeling of corneodesmosome proteins on tape-stripped corneocytes was originally used by Oyama et al. (2010) who studied the lesional skin of two other inflammatory dermatoses with altered terminal differentiation, namely psoriasis and lichen planus. These authors found a diffuse pattern of desmoglein 1 distribution all over the surface of corneocytes from the psoriatic scale, reminiscent of the above-described findings in ichthyosis vulgaris, suggesting that corneodesmosome retention at the ventral/dorsal surfaces of cells might not be pathognomonic but rather related to the relative “maturity” of individual corneocytes in various types of lesions. This point of view seems to be strengthened by the biochemical analysis of the pattern of the cleavage of corneodesmosome proteins in psoriasis, as presented by Simon et al. (2008). The authors detected a near full-length form of corneodesmosin that has not been previously observed in normal SC and altered proteolysis of desmoglein 1, desmocollin 1 and plakoglobin, indicating a reduced degradation of all corneodesmosomal proteins in psoriatic lesions. Studies of dandruff have shown that the persistence of non-peripheral corneodesmosomes is a characteristic feature of the perturbed desquamation seen in this scalp affliction (Singh et al. 2014). The reported observations of the concomitant increased expression of LEKTI-1 and SCCA1 serine protease inhibitors are consistent with the view that the dandruff condition is characterized by an imbalance in protease/protease inhibitor interaction in the SC.

## Acquired forms of corneodesmosome dysfunction and their impact on the SC barrier

Topical application of sodium lauryl sulfate (SLS) detergent to the skin is known to disrupt SC function and is used as a reference in irritation tests in vivo. Skin challenged with 1 % SLS in occlusive patch reacts with early changes of mRNA expression reflecting the up-regulation of pro-barrier elements such as involucrin and transglutaminase 1 and the down-regulation of serine proteases involved in corneodesmosome degradation (Törmä et al. 2008). In soap-induced xerosis, non-peripheral corneodesmosomes also remain undegraded in the upper SC (Rawlings et al. 1994; Rawlings and Voegeli 2013).

Human skin chronically exposed to low temperatures and dry air develops SC dryness characterized by roughness and a papyraceous appearance of the surface, the presence of raised squames and/or scales and irritation, commonly called winter xerosis. In this reactive condition, the persistence of both peripheral and non-peripheral corneodesmosomes in the upper SC has been observed (Simon et al. 2001b). A non-specific character of such corneodesmosome distribution is highlighted by this example.

Epidermal differentiation in palmar/plantar ridged skin represents a particular case, because here corneodesmosomes are not degraded in a pattern known from normal interfollicular epidermis but persist all around the corneocytes up to the surface (Mils et al. 1992). This occurs together with corneocyte accumulation in the ridged horny layer conferring it with more physical resistance. In the case of repeated mechanical stress, the palmar/plantar epidermis reacts with an additional accumulation of the SC, leading to the clinical appearance of calluses. These physiological features well demonstrate the importance of corneodesmosome junctions in the response of the SC to the environment.

## Concluding remarks

Corneodesmosome dysfunction and SC shedding are conditions that clearly affect the epidermal barrier. Impaired barrier function results in an increased cutaneous penetration of environmental allergens and can lead to eczematous reactions. Links between skin inflammation, protease/inhibitor balance, filaggrin processing, the composition and molecular arrangement of the extracellular lipid matrix, corneodesmosome-degradation–dependent desquamation and SC barrier function do indeed exist and should be further investigated.
